# Treatment administered to newborns with congenital syphilis during a penicillin shortage in 2015, Fortaleza, Brazil

**DOI:** 10.1186/s12887-021-02619-x

**Published:** 2021-04-08

**Authors:** Ana Fátima Braga Rocha, Maria Alix Leite Araújo, Melanie M. Taylor, Edna O. Kara, Nathalie Jeanne Nicole Broutet

**Affiliations:** 1 University of Fortaleza-UNIFOR, Av. Washington Soares, 1321, Edson Queiroz, Fortaleza, Ceará CEP 60.811-905 Brazil; 2 World Health Organization, Department of Sexual Reproductive Health and Research, Geneva, Switzerland; 3 US Centers for Disease Control and Prevention, Division of Sexually Transmitted Disease Prevention, Atlanta, GA USA

**Keywords:** Penicillin, Syphilis, Congenital syphilis, Shortage, Treatment

## Abstract

**Background:**

Between 2014 and 2016, Brazil experienced a severe shortage in penicillin supply, resulting in a lack of treatment among some pregnant women and newborns with syphilis and the use of non-evidence-based regimens. This study evaluated all live births in Fortaleza reported with CS in 2015 in order to identify the different therapeutic regimens used in newborns during this period of penicillin shortage.

**Methods:**

A retrospective cross-sectional study design was conducted using manually extracted data from medical chart review of maternal and infant cases delivered in 2015 from all public maternity hospitals in the city of Fortaleza. Data collection occurred from June 2017 to July 2018.

**Results:**

A total of 575 congenital syphilis cases were reported to the municipality of Fortaleza during 2015 and 469 (81.5%) were analyzed. Of these, only 210 (44.8%) were treated with a nationally-recommended treatment. As alternative therapeutic options, ceftriaxone was used in 65 (13.8%), Cefazolin in 15 (3.2%) and the combination of more than one drug in 179 (38.2%). Newborns with serum VDRL titers ≥1:16 (*p* = 0.021), who had some clinical manifestation at birth (*p* = 0.003), who were born premature (*p* <  0.001), with low birth weight (*p* = 0.010), with jaundice indicative of the need for phototherapy (*p* = 0.019) and with hepatomegaly (*p* = 0.045) were more likely to be treated with penicillin according to national treatment guidelines compared to newborns treated with other regimens.

**Conclusion:**

During the period of shortage of penicillin in Fortaleza, less than half of the infants reported with CS were treated with a nationally-recommended regimen, the remaining received treatment with medications available in the hospital of birth including drugs that are not part of nationally or internationally-recommended treatment recommendations.

## Background

Global estimates from the World Health Organization (WHO) showed that, in 2016, approximately 1 million pregnant women were infected with syphilis resulting in over 600,000 congenital syphilis (CS) cases of which 355,000 were adverse birth outcomes including 200,000 stillbirths and neonatal deaths [1]. In 2015, more than 22,800 cases of CS were recorded in countries in Latin America and the Caribbean, corresponding to an incidence rate of 1.7 cases per thousand live births. This rate is mainly related to the cases reported by Brazil, which corresponded to 85% of the records in this region [2].

In Brazil, CS is a mandatory reporting event and recent epidemiological data indicate an incidence rate of 9.0 cases per thousand live births, with the Northeast Region presenting 9.6 cases per thousand live births [3]. These rates are much higher than the elimination threshold established by the WHO, which is less than 0.5 cases for every thousand live births [4] showing that effective prevention measures in general and high-risk populations in Brazil need to be implemented for this goal to be achieved.

Between 2014 and 2016, Brazil, along with 38 other countries, experienced a serious penicillin supply shortage due to manufacturing interruptions and quality assurance issues, resulting in lack of correct treatment among some pregnant women and infants with syphilis and use of non-evidence-based regimens [5]. The Ministry of Health (MoH) recommended the prioritization of benzathine penicillin for the treatment of pregnant women with syphilis and crystalline penicillin for infants with CS. The MoH did not recommend alternative treatment regimens for treatment of syphilis among pregnant women when penicillin was not available. The alternative treatment regimen recommended for infants during the period of penicillin shortages included ceftriaxone 25 to 50 mg/kg, once a day, intravenously or intramuscularly for 10 to 14 days [6]. These alternative treatments for infants were proposed without scientific evidence demonstrating their effectiveness [6].

Fortaleza, State of Ceará, Northeast Brazil has a population of approximately 2.5 million inhabitants, and ten public maternity hospitals. These maternities attend an average of 39,000 births a year and are responsible for 99.4% of the municipality’s CS notifications. Health care is offered through the Unified Health System (SUS), which has a network of services with different levels of complexity. Maternal syphilis rates are high in Fortaleza and CS is a serious problem with detection rate of 23.1 cases per thousand live births [7], much higher than the national incidence rate [3]. During 2014–2016, antenatal care clinics and obstetrics units in Fortaleza experienced different shortage scenarios varying from total stockout to insufficient quantity of penicillin formulations (benzathine, procaine, crystalline) to supply the demand for treatment of pregnant women and newborns. This study evaluated all live births in Fortaleza reported with CS in 2015 in order to identify the different therapeutic regimens used in newborns reported with CS during the period of penicillin shortage.

## Methods

A retrospective cross-sectional study design was conducted using manually extracted data from medical chart review of maternal and infant cases delivered in 2015 from all public maternity hospitals in the city of Fortaleza.

The Brazilian MoH CS definition was used as follows [8]:

- Live born infant whose mother presented, during prenatal care or at the time of delivery, with a reactive non-treponemal test with any titration and reactive treponemal test, and who was not treated or received inadequate treatment.
- Live born infant whose mother was not diagnosed with syphilis during pregnancy, and when it is impossible to perform the treponemal test at the maternity, has a reactive non-treponemal test of any titration at the time of delivery.
- Live born infant whose mother was not diagnosed with syphilis during pregnancy, and when it is impossible to perform the non-treponemal test at the maternity, has a reactive treponemal test at the time of delivery.
- Live born infant whose mother has a reactive treponemal test and a non-treponemal non-reactive test at the time of delivery, without prior treatment.

According to Brazil MOH guidelines in 2015, maternal syphilis treatment is considered inadequate when a treatment other than benzathine penicillin G is used; when the benzathine penicillin G is not administered according to the clinical stage of the infection; when it is not started up to 30 days before delivery; and/or when the partner has untreated syphilis [8].

The medication recommended for use in the treatment of infants or children with CS is penicillin (crystalline, procaine or benzathine). Need for treatment is based on the maternal treatment during pregnancy, the titration of the newborn’s non-treponemal test compared to the maternal, and the newborn’s clinical findings and laboratory tests as follows [8]:

1. Newborns without clinical signs of CS at birth with a non-reactive serum VDRL, should receive benzathine penicillin G, a single dose of 50,000 IU/kg intramuscularly.
2. Newborns with abnormal cerebrospinal fluid (CSF) (reactive CSF VDRL, elevated protein) should receive aqueous crystalline penicillin G 50,000 IU/kg/dose, intravenously, every 12 h (in the first 7 days of life) and every 8 h (after 7 days of life), for 10 days.
3. Newborns with clinical evidence of congenital syphilis and/or reactive serum VDRL, and/or abnormal long bone x-rays and/or changes in the complete blood count should receive aqueous crystalline penicillin G 50,000 IU/kg/dose, intravenously, every 12 h (in the first 7 days of life) and every 8 h (after 7 days of life), for 10 days; or procaine penicillin G 50,000 IU/kg, single daily dose, intramuscularly for 10 days.

Data collection occurred from June 2017 to July 2018. CS case notification forms and hospital medical records were reviewed to collect information on infant treatment. When there were inconsistencies between the records, the data in the medical record was used.

Cases of newborns who died, non-residents in the city of Fortaleza and infant cases whose medical records were not found were excluded. Infants who died were excluded, due to deaths occurring very early (up to 4 days of life), thus treatment regimens could not be adequately accessed. The exclusion of those who did not live in Fortaleza was due to the fact that data from this study were part of a broad project that also intends to collect data on the follow-up of these children for future analysis and publication. The infants who do not live in Fortaleza are followed in their municipalities, which would make access to this data impossible.

Cases who had co-infection with HIV, hepatitis B and C, toxoplasmosis, rubella, cytomegalovirus, congenital herpes virus or zika virus were also excluded due to the possibility of interference in the evaluation of the clinical manifestations and laboratory findings of CS.

The variables analyzed were: timing of mother’s diagnosis, mother’s treatment during prenatal care, gestational age at delivery, baby’s weight at birth, performance and result of the non-treponemal test (VDRL) of peripheral blood and CSF, presence of CSF abnormalities, radiological examination of long bones, changes in the complete blood count, presence of clinical manifestations of CS at birth, and treatment regimen administered to the newborn.

The nationally-recommended treatment scheme for CS comprised any of the three options: 1) aqueous crystalline penicillin G 50,000 IU/kg/dose, intravenously, every 12 h (in the first 7 days of life) and every 8 h (after 7 days of life), for 10 days; 2) procaine penicillin G 50,000 IU/kg, single daily dose, intramuscularly for 10 days; 3) benzathine penicillin G, a single dose of 50,000 IU/kg intramuscularly. Treatments that followed the national guideline regimens for CS, including clinical and laboratorial parameters of mothers and infants, were considered adequate [8]. Only antibiotics administered to the newborns for the treatment of CS were reviewed, those used for other pathologies were not considered.

Newborn exams were considered abnormal based on the following parameters: CSF- reactive VDRL, protein content > 150 mg/dL and/or leukocytes > 25 cells/mm3; Radiography of long bones - presence of involvement in the metaphysis or diaphysis or findings consistent with periostitis, osteitis or osteochondritis; Complete blood count – anemia (hemoglobin - 1 to 3 days of life: < 14.5 g/dL, 7 days of life: < 13.5 g/dL), thrombocytopenia (< 150,000/mm3), leukocytosis and leukopenia (reference values for leukocytes: up to 1 day of life: 9000 to 30,000/mm3, 2 to 7 days of life: 5000 to 21,000/mm3; reference values for neutrophils: up to 1 day of life: 6000 to 26,000/mm3, 2 to 7 days of life: 1500 to 10,000/mm3) [8, 9].

Clinical manifestations at birth were those related to early CS: prematurity (birth with gestational age less than 37 weeks), low birth weight (< 2500 g), hepatomegaly, splenomegaly, skin lesions, jaundice consistent with need for phototherapy and pseudoparalysis of the limbs [8].

The data were analyzed using the statistical software SPSS (Statistical Package for the Social Sciences) version 22. A descriptive analysis was performed using the frequency distribution for the categorical variables and bivariate analysis that applied Pearson’s χ2 test and Fisher’s exact test, establishing a 5% significance level and a 95% confidence interval.

This study was approved by the Research Ethics Committee of the University of Fortaleza (UNIFOR) with opinion number 2.110.189.

## Results

In 2015, there were 575 live births reported with CS in Fortaleza. Of these, 106 (18.4%) cases were excluded from the analysis: nine cases of neonatal death, 26 not residing in Fortaleza, 43 whose medical records were not available and 28 which had co-infection. Records from the remaining 469 newborns were analyzed.

Data on the mothers of these newborns revealed that 390 (83.1%) received prenatal care and of these, 264 (67.7%) were diagnosed with syphilis during prenatal care; 79 (16.9%) women did not receive prenatal care and were diagnosed with syphilis at the time of delivery. All women were considered inadequately treated or did not undergo treatment (Table 1).

**Table 1:** Maternal treatment, performed tests and clinical manifestations in cases of CS. Fortaleza, Ceará, 2015. (*n* = 469)

Variables	Number	Percent
**Maternal**		
**Mother did not attend prenatal care**	79	16.9
**Mother attended prenatal care**	390	83.1
**Mother not diagnosed with syphilis in prenatal care**	126	32.3
**Mother diagnosed with syphilis in prenatal care**	264	67.7
**Maternal treatment in prenatal care**		
Inadequate treatment	204	77.3
Untreated	60	22.7
**Newborn**		
**Newborns with reactive serum VDRL result at birth** (*n* = 467)^a^	367	78.6
**Newborns with serum VDRL titration at birth ≥ 1:16** (*n* = 367)	53	14.4
**Alteration in long bone radiography** (*n* = 139)^a^	10	7.2
**CSF alteration** (*n* = 292)^a^	26	8.9
**Type of CSF alteration** (*n* = 26)^b^		
Reactive CSF VDRL test	8	30.8
Proteins > 150 mg/dL	11	42.3
Leukocytes > 25 cells/mm^3^	10	38.4
**Blood count alteration** (*n* = 362)^a^	159	43.9
Type of Blood count alteration (*n* = 159)^b^		
Anemia	43	27.0
Thrombocytopenia	31	19.5
Leukocytosis	83	52.2
Leukopenia	27	17.0
**Showed some clinical manifestation** (*n* = 456)	199	43.6
**Type of clinical manifestation (***n* = 199)^b^		
Jaundice level requiring phototherapy	116	58.3
Low birth weight	87	43.7
Preterm birth	65	32.6
Hepatomegaly	14	7.0
Skin lesions	10	5.0
Splenomegaly	8	4.0
Limb pseudoparalysis	1	0.5

^a^ Data available for newborns who underwent the examination

^b^ Some newborns had more than one CSF alteration, blood count alteration and clinical manifestation

The result of the newborn’s serum VDRL test was reactive in 367 (78.6%) and for 53 (14.4%) the titration was ≥1:16. A total of 26 (8.9%), 10 (7.2%) and 159 (43.9%) infants had alterations at the CSF exam, in the long-bone radiography and in the blood count (anemia, thrombocytopenia, leukocytosis or leukopenia), respectively. Four hundred and fifty-six (97.2%) newborns had a physical examination recorded in their medical files and 199 (43.6%) showed signs and symptoms of CS (Table 1).

**Fig. 1:**
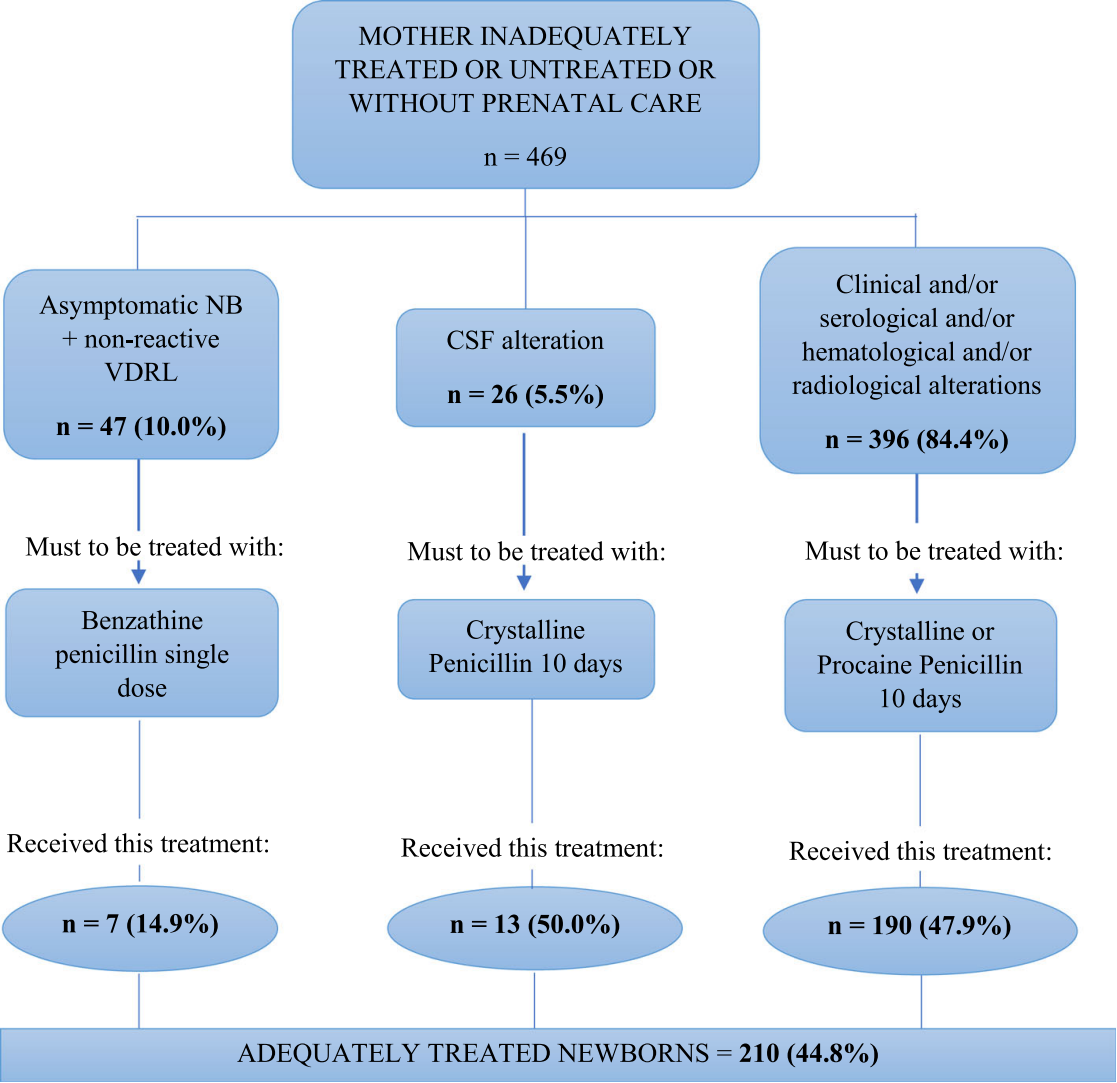
Algorithm for adequate treatment to of newborns (NB) reported with CS. Fortaleza, Ceará, 2015. Source: adapted from: Ministry of Health (BR). Clinical Protocol and Therapeutic Guidelines for Integral Care to People with Sexually Transmitted Infections. Brasília: Ministry of Health; 2015

Of the 469 newborns included in this analysis, only 210 (44.8%) were treated with a nationally-recommended scheme. Of the 47 children who should have received a single dose of benzathine penicillin, 7 (14.9%) received this treatment (Fig. 1) and 40 (85.1%) were hospitalized for 10 days and treated with other intravenous regimens: 12 (30.0%) ceftriaxone, 7 (17.5%) procaine penicillin and 21 (52.5%) received different multi-drug treatment regimens using ceftriaxone combined with penicillin procaine and/or crystalline.

As alternative therapeutic options, ceftriaxone was used in 65 (13.8%) newborns, Cefazolin in 15 (3.2%) and a combination of more than one drug in 179 (38.2%) (Table 2).

**Table 2:** Treatments administered to CS during the penicillin shortage period. Fortaleza, Ceará. 2015. N = 469

Newborns’ treatment	Number	Percent
**Standard scheme**		
Aqueous Crystalline Penicillin G 10 days	187	39.9
Procaine Penicillin G 10 days	16	3.4
Benzathine Penicillin G single dose	7	1.5
**Total**	**210**	**44.8**
**Other schemes**		
Ceftriaxone 10 days	65	13.8
Cefazolin 10 days	15	3.2
Combination of more than one drug (Crystalline Penicillin, Procaine Penicillin, Benzathine Penicillin, Ceftriaxone, Cephalothin)	179	38.2
**Total**	**259**	**55.2**

Multi-drug treatment regimens were used to treat 179 (38.2%) newborns. A majority, 159 (88.8%), received crystalline penicillin as the starting drug for treatment. Each combination presented corresponds to a 10-day treatment schedule (Table 3).

**Table 3:** Other treatment schemes administered to CS combining drugs. Fortaleza, Ceará, 2015. *N* = 179

Initial drug	Complementary drug (s)	Number	Percent
Crystalline Penicillin	+ Procaine Penicillin	134	74.9
	+ Procaine Penicillin + Crystalline Penicillin	6	3.3
	+ Benzathine Penicillin	8	4.5
	+ Ceftriaxone	3	1.7
	+ Procaine Penicillin + Benzathine	2	1.1
	+ Ceftriaxone + Procaine Penicillin	4	2.2
	+ Cephalothin + Procaine Penicillin	2	1.1
Ceftriaxone	+ Procaine Penicillin	17	9.5
	+ Crystalline Penicillin + Procaine Penicillin	3	1.7

Infants with serum VDRL titers ≥1:16 (*p* = 0.021), who had some clinical manifestation at birth (*p* = 0.003), who were born premature (*p* <  0.001), with low birth weight (0.010), with jaundice level requiring phototherapy (*p* = 0.019) and with hepatomegaly (*p* = 0.045) were more likely to be treated with a nationally-recommended penicillin-based regimen (Table 4).

**Table 4:** Alterations identified at birth in newborns reported with CS by treatment scheme, Fortaleza, Ceará, 2015

Variables	Newborn Treatment				***p*** value
	Standard scheme^a^		Other schemes		
	n	%	n	%	
**Serum VDRL result** (*n* = 467)					0.501
Reactive	168	45.8	199	54.2	
Non-reactive	42	42.0	58	58.0	
**VDRL titration** (*n* = 367)					0.021
≤ 1:8	136	43.3	178	56.7	
≥ 1:16	32	60.4	21	39.6	
**CSF alteration** (*n* = 292)					0.174
Yes	13	50.0	13	50.0	
No	97	36.5	169	63.5	
**Long bone radiography alteration** (*n* = 139)					1.000^b^
Yes	3	30.0	7	70.0	
No	46	35.7	83	64.3	
**Blood count alteration** (*n* = 362)					0.155
Yes	61	38.4	98	61.6	
No	93	45.8	110	54.2	
**Showed clinical manifestation at birth** (*n* = 456)					0.003
Yes	105	52.8	94	47.2	
No	100	38.9	157	61.1	
**Preterm birth** (*n* = 469)					< 0.001
Yes	43	66.2	22	33.8	
No	168	41.6	236	58.4	
**Low birth weight** (*n* = 469)					0.010
Yes	50	57.5	37	42.5	
No	161	42.1	221	57.9	
**Jaundice with phototherapy** (*n* = 454)					0.019
Yes	63	54.3	53	45.7	
No	141	41.7	197	58.3	
**Hepatomegaly** (*n* = 456)					0.045
Yes	10	71.4	4	28.6	
No	196	44.3	246	55.7	
**Splenomegaly** (*n* = 456)					0.477^b^
Yes	5	62.5	3	37.5	
No	201	44.9	247	55.1	
**Skin lesions** (*n* = 456)					0.353^b^
Yes	6	60.0	4	40.0	
No	195	44.3	245	55.7	

^a^ Considered when using any of the MoH-recommended treatment schemes: 1) aqueous crystalline penicillin G 50,000 IU/kg/dose, intravenously, every 12 h (in the first 7 days of life) and every 8 h (after 7 days of life), for 10 days; 2) procaine penicillin G 50,000 IU/kg, single daily dose, intramuscularly for 10 days; 3) benzathine penicillin G, a single dose of 50,000 IU/kg intramuscularly

^b^ Fisher’s Exact test

## Discussion

Even in the face of a shortage of penicillin, all newborns reported with CS in Fortaleza during 2015 received some type of treatment. In the setting of limited or no availability of penicillin and unknown effectiveness of other medications used, professionals treated more than half of the newborns with other therapeutic schemes.

Prolonged hospitalizations and excessive use of antibiotics were identified for the vast majority of the newborns of this study who should have received a single dose of benzathine penicillin, making them susceptible to developing adverse reactions, hospital acquired infections, and other complications of hospitalization [10, 11]. Unnecessary hospitalizations also compromised the scarce neonatal beds and increase the costs for the health system.

Unlike other studies [12, 13] carried out in Brazil during periods when penicillin was available, we identified a high proportion of CS cases treated according to the nationally-recommended standard therapy. During 2012 and 2013, prior to the penicillin shortages, Fortaleza had 612 and 663 live births reported with CS and 90,9% and 92,6% respectively were treated with MOH - recommended regimens composed of crystalline, procaine or benzathine penicillin. Of the remainder of cases 8.4% (2012) and 6.8% (2013) had no treatment reported. Only 4 cases in 2012 and 5 cases in 2013 were treated with non MoH-recommended treatment regimens (Unpublished data provided by Fortaleza Health Secretary) compared with 210 (44.7%) in 2015.

The MoH information note describing alternative treatment for CS was published in October 2015, about a year after the shortages began in Fortaleza. This note recommended the use of ceftriaxone for infants with CS in the event of unavailability of penicillin [6]. Therefore, for children born in the period prior to the publication of this note, the choice of treatment was at the discretion of the professionals and the drugs available in the services. This may explain some findings of this study, such as the use of cefazolin and the combination of different drugs.

Although the aim of this study is to evaluate the treatments offered to children reported with CS, it is noteworthy the proportion that did not undergo all the tests necessary for adequate clinical diagnosis. Health authority commitment to ensure access to and availability of adequate testing is essential, to guide appropriate treatment.

In view of the penicillin crisis, neonatologists may have tried to ration the few doses available in the services and prioritize these doses for use in infants with readily apparent clinical and laboratory abnormalities (serum VDRL titers ≥1:16, clinical manifestations at birth, preterm birth, low birth weight, jaundice consistent with need for phototherapy and hepatomegaly), demonstrated by the greater use of standard regimens among these infants. In turn, infants with abnormal CSF, long bone radiography and abnormal blood count findings were not more likely to receive a standard regimen. Though this seems contradictory to provider rationing of penicillin for severe CS cases, these exams are complex procedures within these facilities possibly resulting in delays in access to the results. Physicians may have chosen to treat infants with standard regimens who had obvious clinical manifestations or changes in other tests that were more immediately available.

There are several limitations to this study which demonstrate the missed opportunity to more robustly evaluate the outcomes of treatment regimens among pregnant women with syphilis and infants with congenital syphilis during this period of penicillin shortages. First, as some medical records could not be located, to maximize collection of available data, the authors reviewed data from different data sources including CS case notification forms in addition to medical records. Second, large amounts of data were missing and clinical parameters and test results were not evaluated consistently or were not available for all newborns, thus comparative analyses were limited. It should be noted that no other publications were identified in the peer-reviewed literature whereby data on the use of alternative treatment regimens among newborns with CS were evaluated during a period of penicillin shortages. This made it impossible to compare the results to those of similar cases and settings. Finally, the diagnosis of CS is complex. In order to prevent the possibility of underdiagnosis and to minimize the risk of sequelae in children, WHO, CDC (Centers for Disease Control and Prevention), and Brazil define CS based on criteria that includes the syphilis treatment status of the mother [8, 14, 15] which may include newborns without the infection.

Although the majority of newborns reported with CS were born without obvious clinical signs, it is possible that they may present early and/or late manifestations of CS if the treatment given was inadequate [16–19]. There is ongoing concern related to the follow-up of newborns reported with CS in Brazil during the period of 2014–2016 who received other treatment regimens for which clinical efficacy data are not available. It is expected that these infants received outpatient follow-up and assessment for the need for retreatment when penicillin was available once again [6]. Findings from this analysis were presented to health managers in the city of Fortaleza in 2019 with the aim to reinforce the importance of performing an active search for children who may not have received adequate follow-up. Data from pediatric follow-up were not collected for this analysis but will be evaluated as part of future study.

The efficacy and clinical outcomes following use of alternative treatments provided to newborns with CS are not known. For this reason, comprehensive monitoring of these children is essential to assess their health status in the medium and long terms, as well as the need for possible retreatment [6]. Review of clinical follow-up of these infants will be considered for further study. Considering the possibility of recurrence of the shortage of penicillin [20], research that evaluates efficacy of alternative drugs for the treatment syphilis in pregnant women and newborns is essential.

## Conclusion

These results demonstrate that in 2015, during the penicillin shortage, in Fortaleza, the proportion of newborns reported with CS who were adequately treated was low. Different therapeutic schemes were defined and administered based on their availability, including drugs that are not part of the MoH protocols. The data in this study warn of the need for clinical monitoring of these children who were treated as newborns with alternative, non-evidence-based treatment regimens for CS in order to detect and treat complications from possible inadequate treatment and to identify and evaluate alternative maternal and CS treatment regimens in advance of future penicillin shortages.

## Data Availability

The datasets generated and/or analyzed during the current study are not publicly available as they will still be further analyzed for other publications.

## Abbreviations

CDC: Centers for Disease Control and Prevention
CS: Congenital Syphilis
CSF: Cerebrospinal fluid
MoH: Ministry of Health
NB: Newborns
SUS: Unified Health System (Sistema Único de Saúde)
VDRL: Venereal Disease Research Laboratory
WHO: World Health Organization
